# Nutrient and heavy metal dynamics in the coastal waters of St. Martin’s island in the Bay of Bengal

**DOI:** 10.1016/j.heliyon.2023.e20458

**Published:** 2023-09-27

**Authors:** Md. Jobaer Alam, A.S.M. Maksud Kamal, Md. Kawser Ahmed, Mahfujur Rahman, Mahmudul Hasan, Sad Al Rezwan Rahman

**Affiliations:** a Department of Oceanography, University of Dhaka, Dhaka, 1000, Bangladesh; b Department of Disaster Science and Climate Resilience, University of Dhaka, Dhaka, 1000, Bangladesh; c Department of Geology, University of Dhaka, Dhaka, 1000, Bangladesh; d Bangladesh Reference Institute for Chemical Measurements, Dhaka, 1205, Bangladesh

**Keywords:** Nutrients, Heavy metals, Concentration, Distribution, St. Martin's island, Bay of Bengal

## Abstract

Seasonal variation observations were conducted in the coastal waters of St. Martin's Island in the Bay of Bengal to examine the influence of physical processes and the distribution pattern of nutrients in the ocean water. Pollution evaluation indices, health index and statistical techniques were incorporated to assess the heavy metal contamination. Two seasons, cool dry winter and pre-monsoon hot, were considered for sampling from 12 stations around the island. The Cool dry winter season has higher nutrient concentrations than the Pre-monsoon Hot season. The concentration of nutrients appeared as follows: Silicate > Nitrate > Ammonia > Phosphate > Nitrite. PCA and Pearson's Correlation showed that fresh water from nearby rivers, deep water upwelling, and, in some situations, modest anthropogenic sources are crucial. Hence, low DO and phosphate levels during the pre-monsoon hot season indicate there is a planktonic process like photosynthesis prevailing. The island's north-western and south-eastern regions have higher nutrient concentrations, which may be seasonal and due to wind action. Pb, Cu, As, Cr, Cd, and Zn were also considered to comprehend the island's geo-chemical perspectives and ecological and human health risks. The Pre-monsoon Heavy Metal Pollution Index (HPI) and Heavy Metal Evaluation Index (HEI) demonstrated that some places are much higher than the threshold limit, even though no significantly higher value was detected in the cool winter season. The Nemerow Index, the Total Ecological Risk Index (TERI), indicated that heavy metal contamination was severe to moderate and low to moderate. Finally, Pearson's correlation showed the association between physical and chemical characteristics, similar to PCA and Pearson's correlation for nutrients and heavy metals. Thus, this research may help shed light on the state of the seas around St. Martin's Island. This study may also provide explicit insights for the authority to take the necessary measures to preserve marine ecology and the associated terrestrial ecosystem.

## 1 Introduction

Coastal ecosystems are dynamic and fragile that play a crucial role in supporting biodiversity, providing habitat for numerous species, and offering valuable resources to human populations. Among the numerous threats they face, nutrient enrichment and heavy metal contamination have emerged as major concerns due to their potential to disrupt the ecological balance and compromise the sustainability of these coastal environments. Coastal islands, with their proximity to industrial and urban areas, are particularly susceptible to this repercussion. Inputs from adjoining rivers bring a considerable amount of nutrients and heavy metals that are also naturally occurring [1].

Nutrient contamination is typically associated with excessive fertilizer use in agricultural practices, wastewater discharges, and stormwater runoff, which can result in harmful algal blooms and oxygen depletion, leading to fish kills and other detrimental impacts on aquatic life [[2], [3], [4], [5]]. Nutrients play their role from primary food producers, phytoplankton to zooplankton. Primarily, phytoplankton use nutrients to generate amino acids, proteins, and associated substances that are then consumed by organisms higher up the food chain [6]. But only a minute portion of these nutrients is required for living organisms. Excessive concentration of nutrients in ocean water is alarming for the ecological components as they lessen the biological productivity and disturbs the biogeochemical processes resulting in nutrient enrichment or eutrophication of the coastal water [[2], [3], [4],^7^,^8^]. Eutrophication or nutrient enrichment triggers a series of manifestations, notably cyanobacterial blooms, jellyfish blooms, hypoxia, and ocean acidification. The susceptibility of nutrient enrichment is higher along the coastline. While considering the anthropogenic sources, numerous sources are active to give rise the nutrient concentration in ocean water and sediment. Rapid urbanization, intensive use of fertilizer and pesticides for irrigation, water-based vehicle waste mixing with ocean water, and increased nutrient concentration in water are all contributing factors [9].

Similarly, seawater contaminated with heavy metals can cause changes in the physical, chemical, and biological properties of the water, and can also affect the behaviors and survival of marine organisms, including fish and shellfish that are important for human consumption. Heavy metals accumulate in the coastal region by both geogenic and anthropogenic sources and both point and nonpoint sources are tracked. Weathering and erosion of parent rock that is transported mainly by river are the prime natural source of erosion [[10], [11], [12]]. But in this contemporary industrial era, anthropogenic sources outweigh natural ones. Specifically, the use of pesticides and chemical fertilizers, along with industrial waste and municipal waste that dump into the river, is the most extensive [13,14]. Despite the fact that just a few metals, such as Cu and Zn, are essential for biological functions in both plants and microorganisms, other metals, such as Cd, Cr, As, and Pb, are deleterious over a specific threshold [[15], [16], [17]]. Accumulation of non-biodegradable heavy metals in animal cells and marine plant cells is fatal due to the destruction they inflict on biophysical functions [12,[18], [19], [20]].

Considering the ecological and environmental consequences of nutrient enrichment and heavy metal contamination, there is a demand for a systematic study on St. Martin's Island, the most versatile ecosystem in the Bay of Bengal. This island is very resourceful with enormous biological diversity, i.e., mollusks with 300 species, fish with 150 species, amphibian-5 species, coral-66 species, turtle-5 species, snail-5 species, bird-200 species, mammals-20 species [21]. The islands' ecosystem encompasses rocky and sandy intertidal habitats, rocky subtidal habitats, a wide range of flora and fauna, soft coral habitats, and soft-bottom offshore habitats [22,23]. The geographic location of the island turned the place vulnerable. The study area is situated in close proximity to the mouth of the world's biggest delta system, where numerous rivers connect with the Bay of Bengal. Thus, a considerable amount of sediment passes through the study area. Besides, the input from upstream also carries urban, industrial, and municipal waste that contains higher concentrations of nutrients and heavy metals.

Due to their relevance, studies on the sources and rate of accumulation with respect to spatiotemporal variation of nutrients have become an important research topic in coastal and estuarine ecosystems. Previously, the majority of the research was conducted on temporal and spatial variation in different parts of the world [[24], [25], [26]]. Several studies on some ecological disasters like hypoxia, eutrophication, and algae blooming were carried out. Most explicit research has been conducted on phytoplankton distribution, abundance, and diversity due to nutrient variation in different ecosystems. Some limited approaches for nutrient distribution were taken in the coastal areas of the Bay of Bengal [27,28].

In contemporary times, heavy metals have garnered substantial attention from researchers all around the world for their long-lasting and deleterious effects [[29], [30], [31], [32], [33]]. In the majority of cases, spatial distribution and heavy metal accumulation in marine plants and organism cells were assessed [[34], [35], [36], [37], [38], [39], [40]]. Regionally in Bangladesh, some research endeavors on heavy metal indexing and ecological risk indexing were taken into consideration [41]. But no such study has yet been conducted in the study area.

The prime objective is to draw an evaluation of the dynamics and concentration of nutrients and heavy metals in the seawater of St Martin's Island. Pollution indexes, particularly heavy metal pollution index (HPI), heavy metal evaluation index (HEI), and Nemerow pollution index (NI), along with multivariate statistical approaches, namely principal component analysis (PCA) and correlation analysis, are executed to enumerate the pollution status and provide insight about the probable sources of nutrients and heavy metals in a particular year. This study is critical in order to construct a theoretical and numerical framework for nutrients and heavy metals on St. Martin's Island. This insight empowers us to implement targeted conservation measures, adopt sustainable practices, and advocate for responsible development, all in the pursuit of safeguarding that pristine coastal island for generations to come. Eventually, this will encourage monitoring the concentration of nutrients and heavy metals on the basis of specific and persistent temporal changes in the seawater adjacent to St. Martin's Island.

## 2 Study area

**Fig. 1:**
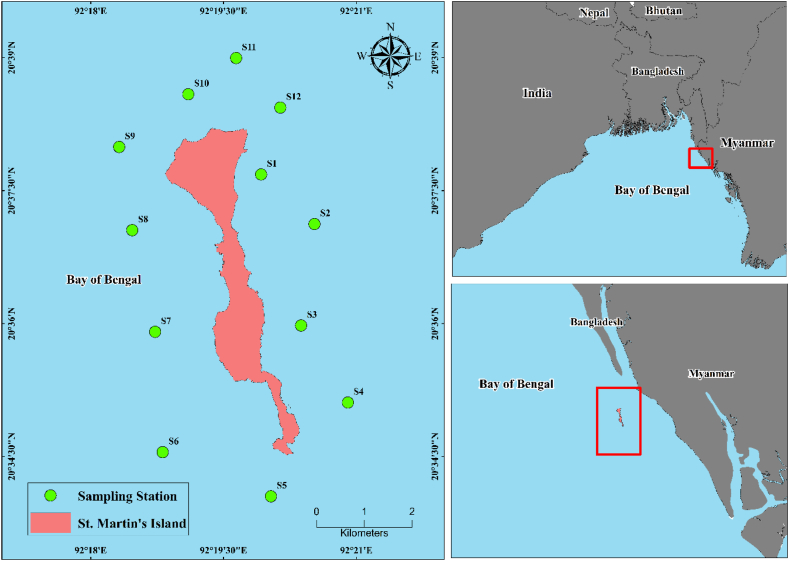
Map showing the study area and the location of the sampling sites, coastal waters of St. Martin's Island.

St. Martin's Island in Bangladesh is an exclusive coral-bearing offshore island, which makes the territory one of the most enticing tourist hotspots. It is located in the vicinity of the Teknaf peninsula in a south-eastern direction from the coast of Bangladesh (Fig. 1). Diagonally the island is about 9 km south apart from Cox's Bazar, Bangladesh and approximately 8 km west from the north-west coast of Myanmar [42]. The island is situated towards the northeast of the Bay of Bengal. Geographically lying between 20°34′ and 20°39′N latitude, and 92°18′ and 92°21′E longitude [23]. The island occupies a total area of 12 km^2^, whereas the terrestrial area is engrossed by 5.9 km^2^ and the rest of the 6.1 km^2^ is a rocky platform extended to the sea [43]. The dumbbell-shaped island is divided into 5 distinct physiographic areas, namely the Uttor Para, Madhya Para, Dakhin Para, Golachipa, and Cheradip areas.

Geologically, the island is a sedimentary continental island, and the climate is mostly governed by the subtropical monsoons [23]. There are three dominating seasons on the island: the Pre-monsoon hot season, Rainy monsoon season, and the Cool dry winter season. During the monsoon season, access to the island is prohibited due to the hostile weather. The island and the nearby vicinity have yearly rainfall fluctuations between 280.50 and 300 mm and more than 90% of the rainfall is received during the monsoon period (https://bmd.gov.bd/). The range of the lowest temperatures on the island that are estimated is between 19.3 and 22.4 °C, while the mean annual maximum temperature is from 30.3 to 33.0 °C [44].

The island is subject to a substantial tidal influence, which is distinguished by regular semidiurnal tides that last for 24 h and 52 min. Because of its rotation, the island experiences a cycle of two high tides and two low tides per cycle. As the tide range around the island is about 1.87 m, the Cheradip area gets cut off from the rest of the island when it submerges during high tide [44].

## 3 Methodology

### 3.1 Surface seawater sampling and analysis

Data collection was conducted during two specific seasons, namely the cool winter of 2020 and the pre-monsoon period of 2021, with the aim of conducting a comprehensive investigation of the peripheral region of St. Martin's Island in the Bay of Bengal. The collection of data for the remaining season was hindered by technical inconveniences like transport and inaccessibility. Water samples were collected from 12 different sampling locations (n = 36). This study quantifies five nutrient parameters, evaluates concentrations for six heavy metals, and measures five physicochemical parameters to gain a holistic understanding of the water quality metrics.

Samples of surface seawater were extracted using Niskin bottle samplers at a depth of 0.5 m below the air-water interface stratum. In accordance with Rice et al. [45], the process was repeated three times to confirm quality assurance and quality control. The entire sampling process during the two seasons was carried out by fishing vessels rented from St. Martin's Island's Jetty. Prior to collecting each sample, the trawler was stopped for about 10–15 min so the ambient conditions could be achieved. To prevent suspended particle matter, samples were filtered using pre-weighted 25 mm Whatman GF/F filter paper and frozen at −20 °C according to the protocols until further analysis. Concurrently, a calibrated multimeter (HI-98194) was employed to measure the temperature (T), pH, salinity, and total dissolved solids (TDS) that were afterward recorded for associated analysis. The accurate concentration of dissolved oxygen (DO) was quantified using a portable DO meter (HQ40d), and samples without bubbles were taken into account. Each time while sampling, a handheld GPS meter was used to track the geographical position of the samples. However, consecutive sampling was interrupted from May to August because of inconvenient weather and the associated logistical assistance required for incorporating sampling collection.

In seawater, dissolved inorganic nitrogen (DIN) consists of nitrate and nitrite ions, dissolved inorganic phosphate (DIP) consists of phosphate ions, and dissolved inorganic silicate (DSi) consists of silicate ions. In the laboratory analysis, the concentrations of nitrate (NO_3_^−^), nitrite (NO_2_^−^), ammonium (NH_4_^+^), phosphate (PO_4_^−^), and dissolved silicate (SiO_4_) were determined by following the standard spectrophotometric procedures using a double-beam spectrophotometer (Shimadzu UV–Visible 1800) outlined by Grasshoff et al. [46]. The study maintained standard operating procedure (SOP) in the laboratory, chemical and safety protocol. Additionally, preparation of the blanks and washing and rinsing of the glassware were done with special precautions.

In conjunction with the objective is the use of Coupled Mass Spectrometry (ICP-MS) for measuring the concentrations of heavy metals, including Pb, Cu, As, Cr, Cd, and Zn, in a lab (Bruker Aurora M90, Bremen, Germany). The laboratory method that was followed in this study was outlined by Kimura et al. [47]. HNO3 and HCl were used to prepare standards and solvents for analytical procedures, both for extraction and acid digestion according to the Standard operating Procedure (SOP). The 100 ppm of transition metal stock standards (Merck, Germany) were used to produce 50 g/L, 20 g/L, 10 g/L, and 5 g/L intermediate standard solutions. Calibration curves were used for analysis of all the heavy metals after each parameter was adjusted for optimal sensitivity and magnitude of the signal.

### 3.2 Heavy metal health and pollution index

#### 3.2.1 Heavy metal pollution index (HPI)

The HPI provides numerical value to track the frequency and degree of heavy metal pollution in collected seawater. The index enables to estimate the extent of heavy metal pollution that impacts human health and the marine environment. The HPI index was calculated by assigning each parameter a rating or weight (Wi) ranging from 0 to 1, which represented the relative significance of multiple quality criteria when taken together. Wi could also be computed by inversely proportional to the parameter's proposed standard (Si) [[48], [49], [50], [51]]. In this study, China's standard for seawater quality is used as the standard concentration limits.

The HPI was calculated using the following formula [52]:

(1) $$HPI=\frac{\sum_{i=1}^{n}W_{i}Q_{i}}{\sum_{i=1}^{n}W_{i}}\text{,}$$

where, Q_i_ and W_i_ are the sub-index and unit weight of parameter i, respectively, and n is the number of parameters considered.

The sub-index Q_i_ is calculated by

(2) $$Q_{i}=\sum_{i=1}^{n}\frac{\left|M_{i}-I_{i}\right|}{S_{i}-I_{i}}\times 100\text{,}$$

where, M_i_, I_i_, and S_i_ are the measured heavy metal concentration, desirable concentration, and standard recommended concentration of parameters i, respectively. The symbol (−) stand for the numerical difference between two values, which ignores algebraic sign.

#### 3.2.2 Heavy metal evaluation index (HEI)

The Heavy Metal Evaluation Index (HEI) is a numerical value that reflects the level of heavy metal contamination in a given area [48,49,[53], [54]]. The HEI is calculated by measuring the concentration of one or more heavy metals in a sample (e.g., soil, water, air), and then comparing the results to established standards. HEI is calculated as follows:

(3) $$HEI=\sum_{i=1}^{n}\frac{H_{c}}{H_{\max}}\text{,}$$

where, H_c_ and H_max_ are the measured concentration and maximum admissible concentration of parameter i. Heavy metal evaluation index is also known as the “Metal index (Mi)” by Tamasi and Cini [55].

#### 3.2.3 Nemerow Pollution Index (NPI)

The Nemerow Pollution Index (NPI) is a composite index that measures the overall level of pollution of water, soil or air in a given area. The NPI enables to track the changes in the level of pollution over time. In this study, the index is taken into account to enumerate the sea surface water pollution and water quality in the vicinity of St. Martin's Island [56,57]. NPI also provides categorizing water quality by the way one specific element affects water [57] and usually calculated by the equation given below:

(4) NPI=[(1n)∑(Ci/Si)]2+[max⁡(Ci/Si)22,

The observed concentration and standard concentration of the heavy metal i are Ci and Si, respectively, and the number of indices is represented by n. The China's secondary seawater quality standard (GB3097-1997) was considered as a reference for assessing water quality. NPI tends to give metal pollution in seawater into six categories: ≤0.5 indicates no pollution, 0.5–0.7 indicates clean, 0.7–1.0 denotes warm, 1.0–2.0 implies polluted, 2.0–3.0 indicates moderate pollution, and >3.0 indicates severe pollution [3,58].

#### 3.2.4 Total ecological risk index (TERI)

The Potential Ecological risk (PER) approach is a method that is used in order to assess the risk that is caused by heavy metal concentration in seawater. PER is equal to the sum of all the elemental risk E^i^_r_. Primarily, the risk (E^i^_r_) for each individual element is determined by multiplying the toxic response factor and contamination factor. The PER is defined as the sum of all the elemental Eir, indicating the sensitivity of the biological community to toxic substances and the overall potential ecological risk.

According to Guo et al. [59], the PER is calculated using the following equations:

(5) $$E_{r}^{i}=T_{r}^{i}\times C_{f}^{i}$$

(6) $$C_{f}^{i}=C^{i}/\;C_{n}^{i}$$

(7) $$C_{d}=\sum_{i=1}^{n}C_{f}^{i}$$

And

(8) $$\text{PER}=\Sigma \;E_{r}^{i}\left(m,i=1\right)$$

where E^i^_r_ represents the ecological risk index for a single element, and T^i^_r_ is the biological toxic factor for each element. C^i^ and C^i^_n_ are the content of the element in samples and the reference value of the element.

The intensity of risk posed by a single heavy metal element on the ecology is categorized into five classes: low risk (E^i^_r_ < 40), moderate risk (40 ≤ E^i^_r_ < 80), considerable risk (80 ≤ E^i^_r_ < 160), high risk (160 ≤ E^i^_r_ < 320), and very high risk (E^i^_r_ ≥ 320) [59,60]. The overall potential ecological risk (PER) in each site, which is calculated as the sum of all elemental E^i^_r_, is further divided into four categories: low risk (PER <95), moderate risk (95 ≤ PER <190), considerable risk (190 ≤ PER <380), and very high risk (PER ≥380) [59]. T^i^_r_ are 5, 5, 10, 2, 30, and 1 for Pb, Cu, As, Cr, Cd and Zn, respectively [59,61].

### 3.3 Statistical analysis

The multivariate statistical technique is a comprehensive numerical method to order, systematize, and extract tangible insights from any extended array of datasets [41,62]. In this study, a combined array of methods, particularly Principal Component Analysis (PCA), and Pearson's Correlation, were taken into account for understanding the relationships among the parameters considered for St. Martin's Island using statistical software, namely SPSS (version 20).

PCA was executed in this study to determine the sources of the nutrients and heavy metals relevant to the variant concentration accumulation and distribution in the studied marine ecosystem. Initially, this is required to define the appropriate dataset for PCA; hence, Kaiser-Meyer-Olkin (KMO) and Bartlett's sphericity assays were conducted. The prime concern of the test is to transform the original variables into a new set of variables (axes) that are referred to as principal components (PCs), which characterize the original variables in a linear combination [63,64]. The correlation between PCs and parameters is drawn by factor loadings, and the most important insights are attributed to the strongest positive and negative loadings [65].

Pearson's correlation is a statistical measure of the linear relationship between two variables. It measures the degree to which two variables are linearly related, and the strength and direction of the relationship [66]. The Pearson's correlation coefficient is always between −1 and +1, where a value of +1 indicates a perfect positive correlation, a value of −1 indicates a perfect negative correlation, and a value of 0 indicates no correlation between the two variables.

## 4 Results and discussion

### 4.1 Seasonal variation of physicochemical parameters, nutrients and heavy metal concentration in coastal waters of St. Martin's island

Physicochemical parameters, nutrients and heavy metals concentration of oceanic water in St. Martin's Island during two seasons, namely Cool winter (CW) and Pre-monsoon (PM) seasons are mentioned in Table 1 and Table 2.

**Table 1:** Measured physicochemical parameters of both seasons (PM and CW) in the studied coastal waters.

Sample ID	Cool Winter (CW)					Pre-monsoon (PM)				
	pH	DO (mg/l)	Temp (°C)	TDS (g/l)	Salinity (psu)	pH	DO (mg/l)	Temp (°C)	TDS (g/l)	Salinity (psu)
S-1	8.10	7.75	24.85	20.16	26.21	7.95	6.26	26.10	23.55	30.62
S-2	8.11	7.95	25.4	20.21	26.37	8.15	6.13	26.20	23.50	30.66
S-3	8.11	7.80	24.61	20.13	26.18	8.22	6.10	26.00	23.62	30.72
S-4	8.13	8.36	24.76	20.15	26.22	8.20	6.49	26.10	23.71	30.85
S-5	8.18	7.59	24.98	20.08	26.15	8.20	6.37	26.20	23.87	31.09
S-6	8.16	6.63	25.14	20.02	26.07	8.13	6.39	26.70	23.82	31.02
S-7	8.14	7.03	24.22	20.16	26.23	8.30	6.48	26.30	23.36	30.39
S-8	8.15	7.82	24.26	20.04	26.09	8.26	6.47	26.30	23.40	30.47
S-9	8.16	7.84	24.22	19.97	25.98	8.18	6.71	26.40	23.36	30.39
S-10	8.14	7.93	24.27	20.09	26.15	8.20	6.60	26.20	23.29	30.32
S-11	8.14	8.00	24.33	20.18	25.98	8.20	6.74	26.10	23.43	30.16
S-12	8.13	8.66	24.16	20.12	26.05	8.14	6.63	26.20	23.29	30.15
Minimum	8.10	6.63	24.16	19.97	25.98	7.95	6.10	26.00	23.29	30.15
Maximum	8.18	8.66	25.40	20.21	26.37	8.30	6.74	26.70	23.87	31.09
Average	8.14	7.78	24.60	20.11	26.14	8.18	6.45	26.23	23.52	30.57
SD	0.02	0.49	0.40	0.07	0.11	0.08	0.20	0.17	0.19	0.31

SD = standard deviation.

**Table 2:** Measured nutrients concentration of both seasons (PM and CW) in the studied seawater sample (All are in mg/l).

Sample ID	Cool Winter (CW)					Pre-Monsoon (PM)				
	Nitrate	Nitrite	Silicate	Ammonium	Phosphate	Nitrate	Nitrite	Silicate	Ammonium	Phosphate
S-1	0.615	0.146	8.995	0.315	0.187	0.312	0.093	7.654	0.115	0.118
S-2	0.627	0.136	8.665	0.287	0.173	0.297	0.097	7.673	0.132	0.103
S-3	0.667	0.139	8.773	0.261	0.131	0.314	0.107	7.704	0.112	0.081
S-4	0.763	0.143	8.641	0.254	0.163	0.384	0.119	7.024	0.137	0.138
S-5	0.568	0.121	8.304	0.255	0.163	0.388	0.112	7.259	0.174	0.133
S-6	0.586	0.144	8.189	0.206	0.154	0.393	0.115	7.881	0.211	0.128
S-7	0.679	0.126	8.536	0.252	0.133	0.399	0.102	7.772	0.218	0.142
S-8	0.722	0.125	8.764	0.224	0.146	0.301	0.097	7.559	0.214	0.138
S-9	0.769	0.138	8.912	0.351	0.236	0.312	0.103	8.346	0.219	0.127
S-10	0.654	0.133	8.795	0.347	0.194	0.305	0.098	8.345	0.186	0.132
S-11	0.781	0.164	9.001	0.313	0.154	0.309	0.087	8.09	0.142	0.105
S-12	0.745	0.173	8.814	0.305	0.184	0.314	0.089	7.78	0.092	0.098
Minimum	0.568	0.121	8.189	0.206	0.131	0.297	0.087	7.024	0.092	0.081
Maximum	0.781	0.173	9.001	0.351	0.236	0.399	0.119	8.346	0.219	0.142
Average	0.667	0.135	8.642	0.267	0.165	0.345	0.105	7.652	0.170	0.123
SD	0.074	0.015	0.253	0.046	0.029	0.041	0.01	0.389	0.046	0.019

SD = standard deviation.

From the analysis, it was found that the pH level ranges for the cool winter and pre-monsoon seasons are 8.10–8.18 and 7.95–8.30, respectively, which is a clear indication of an alkaline nature [35]. The temperature exhibited the highest value in the PM season during March and the lowest value observed during November in the CW season, with a considerable difference in the average temperature between PM and CW that numbered 26.23 ± 0.17 °C and 24.60 ± 0.40 °C. Solar direction, predominant wind actions, and wind-water interaction are the principal determinants of the mean temperature in coastal or island zones or neritic zone [67]. Seasonal mean salinity based on data from 12 sites all across the CW and PM seasons was substantially distinguishable. During the cool winter season, it was 26.14 ± 0.11 psμ, which climbed up to a value of 30.57 ± 0.31 during the pre-monsoon regardless of tidal conditions. A similar time schedule was approximately maintained to avoid tidal effects during sample collection. Compared to the cool winter season, the pre-monsoon season has a comparatively lower level of precipitation and a higher evaporation rate, which could lead to a significant rise in salinity [68]. DO value during the pre-monsoon (PM) season was slightly low (6.45 ± 0.20 mg/L) probably due to the greater abundance of planktonic species, and concurrent limited influxes of fresh water from adjacent areas [[69], [70], [71]]. In contrast, it is was higher during cool winter season (7.78 ± 0.49) might be due to the higher freshwater influx from the adjacent estuaries and higher capacity of cold water to hold dissolve oxygen [69]. A substantially higher magnitude of TDS values was found in the pre-monsoon season than in the cool winter season, tentatively a consequence of sediment and anthropogenic influx from the adjacent run-off that valued 23.52 ± 0.19 whereas the CW showed a mean of 20.11 ± 0.07 g/L.

All the nutrient concentrations exhibited highest mean values during the Cool winter season (Nitrate: 0.667 ± 0.074; Nitrite: 0.135 ± 0.015; Silicate: 8.642 ± 0.253; Ammonium: 0.267 ± 0.046; and Phosphate: 0.165 ± 0.029 mg/L) and during the Pre-monsoon season the values were slightly lower (Nitrate: 0.345 ± 0.041; Nitrite: 0.105 ± 0.010; Silicate: 7.752 ± 0.389; Ammonium: 0.170 ± 0.046; and Phosphate: 0.123 ± 0.019 mg/L). During both seasons silicates and nitrate are the prime contributor of the nutrients followed by ammonium and phosphate, respectively. The driving factors for nutrient concentration of the island are freshwater input [72], sediment inflow [73], nitrate oxidation [74] and in some particular cases upwelling and downwelling of nutrient-rich ocean water [75].

The data presented in Table 3 show that the mean value of heavy metals during the Pre-monsoon hot season (Pb: 76.82 ± 37.91; Cu 27.47 ± 2.78; As: 0.99 ± 0.19; Cr: 3.47 ± 1.92; Cd: 6.36 ± 4.08; Zn: 44.53 ± 12.09 μg/L) was considerably higher in concentration than the mean value of heavy metals during the Cool winter season (Pb: 24.90 ± 9.28; Cu: 23.98 ± 1.71; As: 1.06 ± 0.31; Cr: 3.04 ± 2.23; Cd: 3.72 ± 1.26; Zn: 21.09 ± 11.44 μg/L). The prime reason for higher concentrations of heavy metals during the Pre-monsoon hot season may be due to lower freshwater influx from upstream, a higher evaporation rate, and lower precipitation in the study area. Usually, the heavy influx from the upstream runoff gets mixed with the seawater, which dilutes the concentration; afterward, the heavy water influx carries the heavy metals to the deep sea [17]. However, the sources of the metals are numerous, whereas shipbreaking industries, battery production factories, and frequent building materials, along with lighter ships, cargo ships, and vessel ships, are the prime anthropogenic sources [36,[76], [77], [78], [79]]. In some cases, higher concentrations of As may accumulate in the study area from the weathered parent rock, as if most of the river that ultimately discharged in the Bay of Bengal originated in the Himalayan Mountain belt [36].

**Table 3:** Measured heavy metal concentration of both seasons (PM and CW) in the studied seawater sample (All are in μg/L).

Sample Id	Cool Winter (CW)						Pre-Monsoon (PM)					
	Pb	Cu	As	Cr	Cd	Zn	Pb	Cu	As	Cr	Cd	Zn
S-1	25.52	22.94	1.06	2.07	4.71	49.34	59.28	25.72	0.95	4.58	3.29	47.81
S-2	23.89	25.2	0.96	1.56	3.07	27.87	107.83	27.9	1.09	2.06	6.49	58.04
S-3	19.21	22.93	1.14	2.09	2.89	10.12	80.67	23.67	0.99	1.72	4.69	62.67
S-4	40.41	21.92	1.27	5.76	1.27	11.65	157.59	26.62	0.92	2.72	8.54	51.99
S-5	22.74	24.87	1.58	7.07	1.91	24.76	132.13	29.61	1.34	4.75	11.42	60.75
S-6	13.67	22.98	0.85	1.08	4.68	13.26	85.42	27.65	0.65	3.52	9.8	44.39
S-7	37.19	26.52	0.72	0.68	5.2	36.08	76.56	29.67	1.26	0.88	15.49	45.09
S-8	40.52	21.21	0.59	0.76	3.22	16.92	59.64	23.47	0.91	1.38	6.09	32.51
S-9	20.51	23.09	1.17	1.87	5.09	17.67	48.57	24.67	0.82	2.56	4.53	41.68
S-10	12.86	25.97	0.93	3.79	4.8	8.05	33.53	27.69	0.74	3.89	2.89	38.38
S-11	16.67	26.52	1.66	6.99	4.68	20.78	21.91	29.56	1.13	6.03	0.84	24.43
S-12	25.72	23.7	0.78	2.68	3.21	16.67	58.77	33.51	1.09	7.62	2.31	26.67
Minimum	12.86	21.21	0.59	0.68	1.27	8.05	21.91	23.47	0.65	0.88	0.84	24.43
Maximum	40.52	26.52	1.66	7.07	5.2	49.34	157.59	33.51	1.34	7.62	15.49	62.67
Average	24.9	23.98	1.06	3.04	3.72	21.09	76.82	27.47	0.99	3.47	6.36	44.53
SD	9.28	1.71	0.31	2.23	1.26	11.44	37.91	2.78	0.19	1.92	4.08	12.09

Physicochemical parameters, nutrient concentrations, and heavy metal concentrations in marine ecosystems and environments are closely related to each other. Particularly, salinity and temperature could contribute to lower uptake levels of lead from wetland sediments and enhance heavy metal mobility [80,81]. In particular, aerobic conditions and significantly higher nutrient levels influence the absorption of heavy metals like Pb, Zn. Similarly, anoxic conditions in any marine environment reduce the probability of bioaccumulation of Zn, Pb, and Cu. But DO and nutrient levels had no specific influence on the concentration of Ni [82]. The concentration of DO between 7.0 and 9.0 mg/L facilitates the release of metals from sediment into the water and enhances the bioavailability, whereas at concentrations below 7.0 mg/L, there is little to no metal release into the water. In the study area, higher concentrations of heavy metals during the pre-monsoon season are primarily due to anthropogenic sources other than land-derived sources.

### 4.2 Heavy metal indices

The results of the heavy metal indices for both seasons have been enumerated and displayed in Table 4.

**Table 4:** Calculated heavy metal indices of both seasons (PM and CW) in the studied seawater sample.

Sampling point Index	Pre-Monsoon (PM)				Cool Winter (CW)			
	HPI	HEI	NPI	TERI	HPI	HEI	NPI	TERI
S1	31.38	9.62	1.41	94.04	86.19	6.59	1.04	67.02
S2	140.44	14.48	1.89	162.66	41.40	5.87	0.98	56.09
S3	89.43	11.49	4.04	122.58	39.23	4.92	1.72	49.02
S4	197.90	18.84	4.98	224.02	74.46	6.66	2.51	60.78
S5	268.42	17.99	4.30	218.05	62.45	6.02	0.79	49.08
S6	227.81	13.11	4.31	159.87	90.75	4.77	1.72	53.99
S7	407.45	13.48	4.78	185.84	109.03	7.60	1.03	82.73
S8	131.30	9.35	3.68	109.12	51.29	6.58	2.51	71.11
S9	78.99	8.54	2.50	89.69	98.15	5.62	1.76	63.74
S10	29.57	7.32	2.16	66.53	78.43	5.20	1.94	55.86
S11	87.85	6.08	2.21	43.82	78.80	6.15	2.01	60.35
S12	55.62	10.04	3.01	91.83	39.19	5.78	1.66	57.93
Maximum	407.45	18.84	4.98	224.02	109.03	7.60	2.51	82.73
Minimum	29.57	6.08	1.41	43.82	39.19	4.77	0.79	49.02
Average	145.51	11.69	3.27	130.67	70.78	5.98	1.64	60.64
SD	107.58	3.83	1.17	56.26	22.89	0.77	0.55	9.18

#### 4.2.1 Pollution evaluation indices

##### 4.2.1.1 Heavy metal pollution index (HPI)

The analysis of the results from the HPI index for both the Pre-monsoon hot season and Cool winter season sampling points revealed some significant findings. The Pre-monsoon results showed a range of values between 29.57 and 407.45. The results showed significant variation, with samples 5 and 7 recording the highest values of 268.42 and 407.45, respectively. Although it was noted that considerable number about 50% of the sampling points were beyond the HPI threshold limit of 100 [48,83] during the Pre-monsoon season. However, during the Cool dry winter season none of the sampling points showed higher values than the threshold value of 100 except for the sampling point S7. The significantly higher HPI value in both seasons for S7 might be due to the influx of heavy metals from the adjacent upstream area. Additionally, considerably lower values of HPI in the Cool winter season might be induced by a comparatively higher precipitation rate during the monsoon and higher fresh water input from the upstream drainage system.

##### 4.2.1.2 Heavy metal evaluation index (HEI)

Apart from HPI, the heavy metal evaluation index (HEI) was calculated due to an explicit understanding of the heavy metal concentration around the study area, where the Chinese Seawater Quality Standard were considered as a reference for calculating the H_max_. From the study, it was found that the HEI value varied from 6.08 to 18.84 during Pre-monsoon and from 4.77 to 7.60 during Cool winter (Table 4), with a mean value of 11.69 and 5.98, respectively. The study found that all the sampling points during the Cool dry winter season [48,50] and about 50% of the total sampling points during the Pre-monsoon had medium level of HEI values [48].

##### 4.2.1.3 Nemerow pollution index (NPI)

The Nemerow Pollution Index is incorporated to evaluate the heavy metal contamination level and water quality of a given area. In general, a higher value of the index indicates lower water quality. The study found that during the Pre-monsoon season, the Nemerow Pollution Index ranged from 1.41 to 4.98, with an average value of 3.27. During the Cool dry winter, the Nemerow Pollution Index values fluctuated from warm to moderate pollution degrees, with a range of 0.79–2.51 and an average value of 1.64. In comparison to the NPI critical value, it can be stated that none of the sample stations are significantly contaminated and that all of them are acceptable for the survival and flourishing of marine organisms.

##### 4.2.1.4 Total ecological risk index (TERI)

Excessive concentrations of heavy metals that are not indispensable might interrupt the delicate balance of the ecosystem and cause hazardous issues subsequently. A potential ecological risk index may thus be a considerable tool to delineate the degree of risk propagated in marine ecosystems due to the simultaneous heavy metal deposition in the sediment [84]. The analyzed level of total ecological risk index and potential ecological risk index have been stated in Table 4.

Upon the conclusion of the comprehensive assessment, the range of TERI for both the Pre-monsoon and Cool winter seasons is 43.82–224.02 and 49.02 to 82.73, respectively, which denotes that the samples in the study area lied between the low and moderate risk classes for the Pre-monsoon and all samples lied in the low-risk category for the Cool winter. The descending order of PER for Pre-monsoon and CW season are: S4>S5>S7>S2>S6>S3>S8>S1>S12 > S9>S10 > S11 and S7>S8>S1>S9>S4>S11 > S12 > S2>S10 > S6>S5>S3, respectively. The prime cause for significant level of heavy metal contamination could be due to water-vehicle waste, oil-leakage from lighter ships, cargo ships, fertilizer use on upstream area, waste disposal sites in the coastal area that brought by drainage system [85].

### 4.3 Source identification of nutrients by statistical analysis

In PCA, the number of PCs is equal to the number of original variables, and each PC incorporates all of the surface water quality metrics [17,86]. According to the principle of PCA, only those eigenvalues that are ≥1 is retained to construct the relevant dataset [87]. In this study, a scree plot was constructed using eigenvalues to show the number of PCs that were significant. The result of PCA for both seasons are showed in Table 5.

**Table 5:** The principal component analysis of observed physicochemical parameters.

CW				PM	
Variable	PC1	PC2	PC3	PC1	PC2
pH	−0.439	**−0.787**	0.057	0.107	**0.644**
DO	**0.599**	0.294	0.300	−0.190	**0.638**
TDS	0.210	**0.884**	−0.189	**0.875**	−0.367
Salinity	−0.284	**0.840**	0.404	**0.919**	−0.316
Nitrate	**0.706**	−0.225	−0.370	**0.820**	0.298
Nitrite	**0.603**	−0.042	**−0.622**	**0.913**	0.082
Silicate	**0.919**	0.105	0.008	**−0.668**	0.308
Ammonium	**0.812**	−0.144	0.395	0.270	**0.802**
Phosphate	**0.550**	−0.480	0.560	0.452	**0.738**
Eigenvalue	3.346	2.507	1.285	3.889	2.435
% Variance	37.182	27.861	14.277	43.212	27.056
Cumulative %	37.182	65.042	79.319	43.212	70.268

Analyzing the physicochemical parameter and nutrient concentration, 3 PCs for the Cool winter season and 2 PCs for the Pre-monsoon seasons were identified. During the Cool dry winter season, 3 PCs were considered with a cumulative variance of 79.319% with a total eigenvalue of 7.138. For the Pre-monsoon seasons, 2PCs contributed about 70.268% of total variance, with a total of 6.315 eigenvalues. During the cool dry winter, PC1 contributed to about 37.182% of the total variance, which signify that the contributing nutrients are the most influential. During the cool winter season, silicate (0.919), ammonium (0.812), nitrate (0.706), nitrite (0.603), DO (0.599) and phosphate (0.550) showed positive and higher loading. The output data suggests that the nutrients may be accumulated from similar sources of origin that are attributed to the oceanic water of St. Martin's Island. Significantly higher loadings for this association (PC1) reflect the heavy influx of freshwater into the marine environment during cool dry winter, increasing nitrogenous nutrients, silicates, and DO levels. Furthermore, significant loadings of silicate, phosphate, and DO indicate the presence of diatoms and other planktonic species that thrive in phosphate and silicate-rich environments [88]. That finally contributed to improving the state of dissolved oxygen [72]. During cool dry winter, plankton communities proliferate, which may contribute to the above conditions [89,90]. In the case of PC2, the parameters, particularly, TDS (0.884), salinity (0.840), and pH (−0.787) showed significant loading. Salinity declined during cool dry winter season is a result of dilution of coastal water from heavy rainfall during monsoon and influx of freshwater from the Meghna, Matamuhuri, and Bakkhali estuaries of Bangladesh and as well as influx from upstream of Myanmar region. In PC3, the parameter nitrite (−0.622) shows significant negative loading which indicate the anthropogenic or upwelling from the deep water. Additionally, higher negative loading may be caused by ammonia from nitrogen being oxidized to yield nitrite in the studied region [74].

From the analysis of the parameter's value in the Pre-monsoon season, considerable discrepancies in nutrient sources were identified. In the PC1 during the Pre-monsoon season, parameters like salinity (0.919), nitrite (0.913), TDS (0.668), nitrate (0.820), showed significant positive loading, whereas silicate (−0.668) showed significant negative loading. The higher loading of salinity and TDS could be linked to the lack of rainfall and increased evaporation, as well as to the dominance of neritic water [74]. Higher loadings of nitrite, nitrate, and silicates were induced by high salinity that might be caused due to active oceanic processes, primely upwelling, vertical mixing through cyclones, or Ekman pumping [75]. Phytoplankton activities, particularly elevated photosynthetic activities and predominating influence of neritic water, are those prevailing reasons for reduced levels of nutrient during Pre-monsoon season [90,91]. For PC2, parameter ammonium (0.802), phosphate (0.738), pH (0.644), and DO (0.638) show high positive loadings. High ammonia and phosphate loadings during the Pre-monsoon season suggest the occurrence of minor coastal upwelling and dilution of ocean water [72]. However, salinity showed no significant load with ammonium and phosphate in PC2 but their concentration increased slightly with the increasing trend of salinity during Pre-monsoon [75,88]. The large differences seen may be caused by processes like phosphate adsorption and desorption and the buffering effect of sediment in different environmental conditions [91]. Ammonia showed high loading during Pre-monsoon period that might be due to the anthropogenic input from the adjacent land area [72].

### 4.4 Source identification of heavy metals by statistical analysis

The Principal Component Analysis for the Cool winter season of heavy metals in the seawater samples is shown in Table 6.

**Table 6:** The principal component analysis of heavy metals in the seawater samples for the Cool winter season and pre-monsoon season.

Variable	Cool winter season (CW)			Pre-monsoon season (PM)	
	PC1	PC2	PC3	PC1	PC2
Pb	−0.443	−0.602	0.546	0.783	0.337
Cu	0.419	0.709	0.204	−0.383	0.862
As	0.924	−0.037	0.185	0.188	0.793
Cr	0.934	−0.207	0.162	−0.748	0.485
Cd	−0.288	0.881	−0.092	0.77	0.337
Zn	−0.227	0.359	0.85	0.856	0.022
Eigenvalue	2.231	1.815	1.131	2.68	1.83
% Variance	37.181	30.257	18.85	44.65	30.56
Cumulative %	37.181	67.438	86.288	44.65	75.21

From the PCA analysis of heavy metals in the study area in cool winter and pre-monsoon season portrayed significant insights about the source and accumulation of heavy metal [49,92]. The analysis showed 2 PCs for pre-monsoon season and 3PCs for cool winter season. During the pre-monsoon season, 2 PCs were considered with a cumulative variance of 75.21% with a total eigenvalue of 4.51. For the cool winter season, 3PCs contributed about 86.288% of total variance, with a total of 5.177 eigenvalues.

Around 37.181% of the variance was attributed to PC1, which accounted for the majority during the cool winter season. The loadings for PC1 indicate that Pb has a negative correlation (−0.443) with this principal component, whereas the other variables, namely Cu, As, Cr, Cd, and Zn, have positive correlations. This suggests that Pb has an inverse relationship with the other variables and is therefore not a major contributor to PC1. PC1 might be caused by the leaching of metals carried by the combined sediment of the upstream water inflow [49,93]. The loadings for PC2 indicate that Cd has the highest positive correlation (0.881) with this principal component, followed by Cu (0.709) and Zn (0.359). Cd, Cu, and Zn might be propagated from anthropogenic source like shipping repairing sites, corrosive plating of ships and lighter ships [49,50,79]. The loadings for PC3 indicate that Zn has the highest positive correlation (0.85) with this principal component, followed by Cu (0.204) and As (0.185), denoting atmospheric exposure that dispersed from the nearby industrial region into the seawater [94].

Similarly, for the pre-monsoon season, PC1 accounts for 44.65% of the total variance and PC2 accounts for 30.56% of the total variance, which contributed about 75.21% of the total variance. The simulated dataset showed that the loading of Pb on PC1 is 0.783, which means that Pb is strongly associated with PC1. Similarly, Cu and Cr have a negative loading on PC1 (−0.383), which means that they are negatively associated with PC1. A significantly higher negative loading of Cr (−0.748) might be due to the water influx transported by the river system to the Bay of Bengal [17]. In the case of PC2, all the components have positive loading, where Cu and As contributed the highest loading of 0.862 and 0.793, respectively. This signifies the uniform source, which might be propagated due to mobilization of metal induced by water flushing [50,93].

### 4.5 Correlation analysis for physicochemical parameters and nutrients

The origin, connection, and movement of physicochemical parameters may be inferred from their correlations [95]. Table 7 shows the correlation coefficient of nine (9) physicochemical parameters and nutrients of the surface seawater for two different seasons.

**Table 7:** Correlation analysis of the studied physicochemical parameters and nutrients.

	pH	DO	TDS	Salinity	Nitrate	Nitrite	Silicate	Ammonium	Phosphate
**CW (n = 12)**									
pH	1								
DO	−0.307	1							
TDS	**−0.688**	0.296	1						
Salinity	**−0.524**	0.174	**0.602**	1					
Nitrate	−0.054	0.467	−0.015	−0.490	1				
Nitrite	−0.330	0.018	0.250	−0.429	0.464	1			
Silicate	**−0.533**	**0.538**	0.233	−0.217	**0.613**	0.432	1		
Ammonium	−0.220	0.373	0.062	−0.209	0.329	0.325	**0.723**	1	
Phosphate	0.100	0.217	−0.398	−0.282	0.191	0.172	0.380	**0.764**	1
**PM (n = 12)**									
pH	1								
DO	0.265	1							
TDS	−0.172	−0.319	1						
Salinity	−0.138	−0.466	**0.943**	1					
Nitrate	0.241	0.141	**0.604**	**0.584**	1				
Nitrite	0.213	−0.128	**0.749**	**0.826**	**0.728**	1			
Silicate	−0.009	0.129	**−0.616**	**−0.609**	−0.459	−0.481	1		
Ammonium	0.471	0.148	−0.035	0.093	0.345	0.304	0.294	1	
Phosphate	0.248	0.394	0.085	0.201	**0.534**	0.387	−0.152	**0.746**	1

In Cool winter, DO and nitrate shows a strong positive correlation with silicate. Significant positive correlation between DO and silicate; silicate and nitrate suggest their sources of origin may be similar. For the observed higher values during the Cool dry winter, the discharge of fresh water from the Naf River estuary rich in silicate could be responsible. Silicate values showed poor correlation with salinity whereas significant correlation between DO and silicates indicates that land derived weathered silica rich fresh water might be the main sources of silicates in the present studied coastal region [88]. In addition, high silicate concentrations suggest that nutrients were transported mostly through land drainage [75]. Furthermore, silicate had a significant positive correlation with ammonium and it showed a strong positive correlation with phosphate which indicates weathering of rock silicates that dissolve alkali metal phosphate, which are transported to coastal waters, can also contribute to increased values [72].

Significant strong positive correlation among salinity and the nutrients were observed during pre-monsoon season. The correlation between nutrients and salinity reveals the sources of nutrients in the coastal water. The strong correlation between nutrients and salinity indicates vertical mixing, coastal upwelling, and Ekman pumping in the pre-monsoon period [75]. Whereas decreasing salinity with increasing nutrients concentration are indicative of riverine sources [75,89].

### 4.6 Correlation analysis of heavy metals in the seawater samples of St Martin's island

Pearson's Correlation during cool winter and pre-monsoon season is showed in Table 8.

**Table 8:** Correlation analysis of heavy metals during the cool winter and Pre-monsoon season.

	Pb	Cu	As	Cr	Cd	Zn
Cool winter						
Pb	1					
Cu	−0.377	1				
As	−0.326	0.261	1			
Cr	−0.158	0.286	0.863	1		
Cd	−0.4	0.393	−0.257	−0.422	1	
Zn	0.233	0.205	−0.017	−0.176	0.285	1
**Pre-monsoon**						
Pb	1					
Cu	−0.004	1				
As	0.245	0.471	1			
Cr	−0.264	0.674	0.126	1		
Cd	0.601	0.081	0.319	−0.525	1	
Zn	0.71	−0.334	0.178	−0.48	0.468	1

Pearson's correlation analysis was conducted among six distinct heavy metals and the results are presented in Table 8. This analysis aimed to provide a comprehensive understanding of the sources and associations among heavy metals by examining their correlation coefficients.

During the pre-monsoon period, a significant positive correlation was observed between Pb and Zn (0.710), as well as between Pb and Cd (0.601). These strong correlations indicate a higher level of association or a common source between Pb and these metals. Additionally, Cr had a moderately negative correlation with Pb (−0.264) and a strong positive correlation with Cu (0.674) which suggests that Cr and Cu have different sources. Furthermore, Cd had a moderate positive correlation with Pb (0.601), indicating that Pb and Cd have a unique source to some extent that might be battery production industries and ship cutting industries on the adjacent coastal area [8,32,33,60,96].

In the cool winter season, a strong correlation was found between As and Cr (0.863) suggesting similar association. During and immediately after the monsoon season, a negative correlation of −0.377 was found between Cu and Pb. This suggests that an increase in Cu concentration corresponds to a decrease in Pb concentration. The negative correlation between Cu and Pb could be due to the fact that these two metals have different sources and associations in the environment. Overall, it was observed that there were fewer sources of heavy metals accumulation during the pre-monsoon than during the cool winter season, which is also indicative of the impact of fresh water intake through the nearby estuaries that carries significant amounts of debris and effluents [17].

### 4.7 Relationship among physicochemical parameters, nutrients, and heavy metals

The intricate interplay among nutrients, heavy metals, and physicochemical parameters in aquatic ecosystems constitutes a dynamic nexus of environmental interactions that profoundly shape water quality and ecosystem health. Nutrients, encompassing essential elements like phosphorus and nitrogen, serve as fundamental drivers of biological productivity, fueling the growth of aquatic plants and algae [97,98]. However, this heightened productivity can inadvertently amplify the uptake and accumulation of heavy metals within aquatic organisms due to their affinity for organic matter [99,100]. The physicochemical parameters of water, including pH, temperature, dissolved oxygen, and conductivity, exert pivotal control over the fate and behavior of both nutrients and heavy metals. Fluctuations in pH levels can influence metal solubility, impacting their mobility and bioavailability [101,102]. Concurrently, these parameters can regulate [103,104] this intricate web of relationships underscores the need for comprehensive and site-specific investigations to unravel the complexities of nutrient-metal-physicochemical interconnections, facilitating informed management strategies to preserve and safeguard aquatic ecosystems.

## 5 Conclusions

Observations of the seasonal variation of physicochemical parameters and multiple statistical analyses in the oceanic water of St. Martin's Island suggest that physical processes, such as heavy influx from adjacent run-off, upwelling of deep water, and elevated precipitation, have a substantial impact on nutrient accumulation and distribution. The study also found that the concentration of nutrients is attributed a higher influence and concentration rate during the cool winter season than the pre-monsoon season, which is caused by the least amount of rainfall and higher evaporation rate during pre-monsoon. Statistical analysis, particularly PCA and Pearson's correlation matrix, also suggests that there might be some contribution from anthropogenic non-point sources that is also considerable. As silicate has the highest contribution to the nutrient concentration, which is found to be the result of sediment influx from terrestrial drainage, there is a significant correlation regarding source with phosphate and ammonia. It can be confirmed that all the nutrients contributed from fresh water input from the rivers. The low concentration of DO suggests there is significant planktonic physical process like photosynthesis during pre-monsoon. A crucial insight from the spatial distribution delineates that the area towards the Naf estuary and Meghna estuaries has a higher accumulation rate of nutrients in comparison to other parts of the island. Exceptions were estimated for ammonia, phosphate, and nitrate during pre-monsoon that exhibit higher concentrations towards the deep sea, which might be caused by upwelling of nutrient rich deep water. Besides, Statistical analysis of heavy metals, including Pb, Cu, As, Cr, Cd and Zn indicates that these metals are accumulating greater due to major anthropogenic activities around the island. The heavy metal indices like the Heavy Metal Pollution Index (HPI) showed that about 50% of total sampling locations only in the pre-monsoon season were beyond the threshold limit (>100), which is totally not similar to the cool winter season. The seawater of the island is revealed to be moderate according to the Heavy Metal Evaluation Index (HEI), and 59% of the total sampling site is polluted according to the Nemerow Index in comparison among the sampling stations but according to the value of NPI, no sample site is significantly contaminated yet and all of them are suitable for the living of marine organism. Total Ecological Risk Index (TERI) categorized that seawater of the island fluctuates from low to moderate risk category. The overall observation found in the study is that the accumulation rate of nutrients and heavy metals in the water of the island is significant, which is primarily accelerated by the fresh water influx that brings industrial and domestic waste from the upstream area. Risks to the environment and human health of St Martin's Island are currently sparse and demand to be addressed; else, significant environmental degradation may become prominent. Given the fact that, long-term monitoring could provide more comprehensive yet exact information on the ecological and environmental changes caused by nutrients and heavy metals. Sustainable management could benefit from studying biodiversity, productivity, ecosystem health, and human health concerns.

## Funding

This research was jointly funded by the Bangladesh Bank and the faculty of Earth and Environmental Sciences, University of Dhaka.

## Data availability statement

Data will be made available on request.

## CRediT authorship contribution statement

**Md Jobaer Alam:** Writing – original draft, Validation, Resources, Project administration, Methodology, Investigation, Funding acquisition, Formal analysis, Data curation, Conceptualization. **A.S.M. Maksud Kamal:** Writing – review & editing, Supervision, Methodology, Conceptualization. **Md Kawser Ahmed:** Writing – review & editing, Supervision, Methodology, Conceptualization. **Mahfujur Rahman:** Writing – original draft, Visualization, Software. **Mahmudul Hasan:** Writing – review & editing, Visualization, Validation, Software, Formal analysis. **Shad Al Rezwan Rahman:** Validation, Software, Resources.

## Declaration of competing interest

The authors declare no conflicts of interest.
